# Perception of coronavirus disease (COVID-19) and its relationship with coping strategies and perceived social support in iranian people: a descriptive correlational study

**DOI:** 10.1186/s12912-023-01276-y

**Published:** 2023-04-07

**Authors:** Nasrin Elahi, Mojtaba Miladinia, Javad Zarei, Saeed Ghanbari

**Affiliations:** 1 Department of Nursing, Nursing Care Research Center in Chronic Diseases, School of Nursing, Ahvaz Jundishapur University of Medical Sciences, Ahvaz, Iran; 2 Student Research Committee, Ahvaz Jundishapur University of Medical Sciences, Ahvaz, Iran; 3 Department of Health Information Technology, School of Allied Medical Sciences, Ahvaz Jundishapur University of Medical Sciences, Ahvaz, Iran; 4 Biostatistics Department, Ahvaz Jundishapur University of Medical Sciences, Ahvaz, Iran

**Keywords:** Illness perception, COVID-19, Coping strategies, Social support, SARS-CoV-2, Nursing

## Abstract

**Background:**

Perception of the threatening disease leads to coping behaviors that can affect the treatment process. Social support can be one of the factors influencing the perception of the disease and coping strategies. Our study aimed to determine the perception of the disease, its relationship with coping strategies and social support in COVID-19 patients in Iran.

**Methods:**

This cross-sectional study was conducted on 1014 patients who were hospitalized during October 2020 to May 2021 through multi-stage sampling method. The data-gathering instruments consisted a demographic information checklist, and standard questionnaires including disease perception, social support, and coping strategies. Correlation coefficient, multiple linear regression model, and simple linear regression model were used for data analysis.

**Results:**

The mean age of the participants was 40.87 ± 12.42 and the majority of them were female (67.2%(, married (60.1%), and had relatives who had COVID-19 (82.6%). There was a significant inverse relationship between variables (identity, outcomes, emotional expressions etc.) and social support (> 0.01). Also there was a significant direct relationship between variables (self-control, therapeutic susceptibility etc.) and the coping behavior (p < 0.05). There was an inverse relationship between the variables (outcomes, self-blaming, sex, etc.) (P = 0.0001) and a direct one between the variables (education, disease phase, etc.) and perceived social support (P = 0.004).

**Conclusions:**

These results show the importance of promoting positive coping strategies and social support in the face of large-scale health crises. The knowledge of nurses about the results of this study, who are responsible for the care and education of the patient, can be effective in the length of hospitalization and reducing costs.

## Introduction

One of the crises of 2020 was the prevalence of Coronavirus (SARS-CoV-2) that spread quickly causing many infections and deaths over all of the countries in the world. By 18 February 2022, over 418 million confirmed COVID-19 cases and over 5.8 million deaths have been reported globally. In Iran, over 6.8 million people had been infected by February 2022, from which 134,420 people died [1]. Numerous studies conducted on patients of COVID-19 have shown that changes in the process of life can cause psychological disorders such as anxiety, fear, depression, emotional changes, amnesia and stress disorders that can infect all age ranges [2, 3]. Thus, it has been reported that the COVID-19 pandemic causes negative effects on mental health in the general population, and especially in patients with the diagnosis/suspicion of COVID-19 [4, 5]. Researchers noted that there is always a mutual relationship between stressor factors (disease) and the person experiencing it, so based on the person’s cognitive biases, it may be considered challenging, threatening or damaging [6, 7]. In the word, the patients’ response to the disease or health risk factors, results in the formation of a common feeling in patients which is referred to as disease perception. Perceptions of disease affects health related behaviors and dealing with the disease, which can eventually affect the outcome of the disease [7, 8]. Neto et al. reported that perceptions of disease are important predictors of behavioral and emotional responses in many disorders [9]. According to the common sense model of Self-Regulation (CSM) that is a widely used theoretical framework that explicates the processes by which patients become aware of a health threat, navigate affective responses to the threat, formulate perceptions of the threat and potential treatment, patients create cognitive representations based on the disease information, and then these beliefs can influence their coping strategies. Hence, disease perceptions and coping strategies are considered as two important mediators between disease status and disease outcomes [10, 11]. In other words, in this study, based on the model of common sense and self-regulation, the perception of illness is an independent variable, which is the result of behaviors, coping strategies, and social support, which are considered as dependent variables.

Coping strategies are a set of psychological responses to the perception of the threat with the aim of preventing or reducing threats, injuries and losses or reducing the discomfort caused by them. Using coping strategies leads to related outcomes and emotional responses such as anger, fear, sadness, disgust, surprise, anticipation. The researchers emphasize that perception of a sickness and the coping behaviors people choose would affect mental health [8, 12]. One of the important sources of coping with stressful situations is the amount of protection and attention received from the relatives [13–15]. Chew et al. reported that patients seeking social support during a pandemic experience less fear/anxiety and depression. Moreover, coping methods such as problem-solving, seeking social support, distraction/denial/avoidance, and positive thinking have been used to reduce psychological distress symptoms [16]. Uchida et al. emphasis that social support serves as an independent and predictor variable for all aspects of health and culture is the factor influencing it [17].

Despite the rapid and ever growing epidemic of COVID-19 throughout Iran and the rest of the world, social isolation from it and sensitivity of mental issues, it is necessary to attend to the sources of service providers and social supports and to identify strategies patients use to cope with the illness and to gain an understanding of how patients’ perceptions of illness and social support are effective in choosing coping strategies that can improve the psychological adjustment. Therefore, the aims of this study were to determine the patient’s perception of the disease and its relationship with demographic characteristics, coping strategies and social support among Iranian people infected by Coronavirus.

### Study context

The study site (Khuzestan province, southwest of Iran) is known for its ethnic diversity, so their social perception is different. This indicates the existence of different cultures and customs between them. But the common denominator among the majority of these tribes is the belief in life as “extended family.” This means that financial and moral support is common among family, friends and people. Khuzestan’s population is predominantly Shia Muslim, but there are small minorities of Sunni Muslim, Christian, Jewish and etc.

## Methods

### Design and setting

This descriptive-correlational study was carried out from October 2020 to May 2021. It was performed in 9 educational hospitals affiliated to Ahvaz Jundishapur University of Medical Sciences. Patients from 10 cities in 5 regions (North, South, East, West and Center) of Khuzestan province, southwest of Iran were included. In each city of province, according to the population, 1 to 3 hospitals were allocated as treatment centers for COVID-19 patients. In these centers, in addition to acute treatment, patients were admitted to special COVID-19 wards.

### Participants

The statistical population included all COVID-19 patients in Khuzestan province who were registered in the first and second couriers by the COVID-19 patients’ registration center of Ahvaz Jundishapur University of Medical Sciences. The required sample size was estimated to be 952 with a 95% confidence interval, P2 0.10 and £ = 0.05p2. With the possibility of dropping 15% of the samples, the sample size was estimated to be 1095.

$$\begin{array}{l}{\rm{N}} \ge \left( {\frac{{2 - 2{\rho ^2} + \varepsilon }}{\varepsilon }} \right)(k + 1) = \\\left( {\frac{{2 - 2 \times 0.2025 + 0.010125}}{{0.010125}}} \right) \times (5 + 1) = 951.158 = 952\end{array}$$

**Fig. 1:**
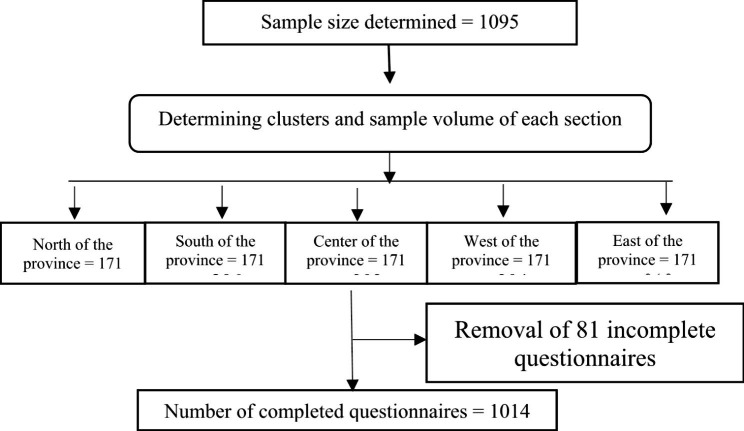
Sampling process

The participants were selected using the available multi-level cluster sampling method based on the inclusion and exclusion criteria (Fig. 1). Inclusion criteria were: 1) patients with confirmed COVID-19 (positive RT-PCR test; 2) over 18 years of age; 3) patients who were hospitalized; 4) at least two weeks passed from getting infected. Exclusion criteria were: (1) patients with suspected COVID-19; (2) outpatients; (3) Incomplete questionnaires. All patients consciously agreed to participate and they provided their written informed consent to participate in this study.

### Data collection

The research questionnaire consisted of four sections that were completed by the participants. The first part was related to the participants’ personal qualities. In the second part, “multidimensional scale of perceived social support” (Zimet et al. 1990) which used social support resources, including family, friends and important people in one’s life was used. This tool is a self-administered scale of twelve options including three sub-scales, in four options. The total score of this scale was 60 points. The Cronbach’ s alpha of the scale as well as the sub-scales were from 0.85 to 0.91 and the reliability through retest was reported to be from 0.72 to 0.85 [18]. This tool in was also reliable in Iranian population [19, 20].

The third part was related to the study of coping strategies in COVID-19 patients which was performed using standard questionnaire and short sheets of coping strategy questionnaires. The questionnaires were designed by Carver et al. and included 28 items and 14 scales (self-distraction, active confrontation, drug use, using social support, using supply tools, behavioral disengagement, relieving, positive regulatory, planning, humor, acceptance, religion, self-blaming). The scores are from 4 (I never do this) up to 1 (I always do this) [21]. Carver et al. regarded the coping flexibility hypothesis (compatible, incompatible) as useful but insufficient. In Iran, this questionnaire has been used in several studies and its validity and reliability has been reviewed and confirmed by Hashemian et al. [22].

In the fourth part, Brunet et al. (2006) modified illness perception questionnaire (IPQ-R) was used in order to measure the perception of disease, which included 9 aspects. This questionnaire measures the identity (attribution of symptoms), consequences (the impact of the disease on their lives), timeline-acute/chronic (duration of the illness), personal controllability (their impact on the disease), coherence of the disease (how much they accept the disease understand), treatment control (the degree of compliance of the disease for medical interventions), assesses time-course (whether the course of illness is constant or cyclical), emotional representations (emotional impact of illness), and causes (illness). The Cronbach’s alpha coefficient was calculated 0.79 to 0.89 for the range of tools used in the study [23]. This questionnaire was translated by Rahimi et al. in hemodialysis patients to Persian and its validity was reported as a suitable criterion [24].

In this study, Cronbach alpha coefficient method was used to measure the reliability of the tools. The results were 0.87 for the scale of perception of the disease (IPQ - R), 0.88 for coping strategies (brief coping), and 0.77 for health-related social support (HRQOL).

### Data analysis

The SPSS_20_ was used to analyze the data. Descriptive statistics were presented for quantitative variables using mean and standard deviation and for qualitative variables using frequency and percentage. Deductive statistics (correlation coefficient, multiple linear regression model, and simple linear regression model) and a significant level were considered 0.5.

## Results

### Participant characteristics

The majority of participants were female (67.2%) and married (60.1%). More than 90% had intermediate and higher education (78.7% and 16%, respectively). And also, 82.6% (n = 848) of the participants, had relatives who had COVID-19 (Table 1).

**Table 1:** Participant characteristics

Variables	Percentage	Number
**Sex**		
male female	32.8% 67.2%	333 681
**Age**		
24–44 41–60 > 60	16.2% 69.5% 14.2%	165 705 144
**Marital status**		
single married divorced widowed	3.9% 60.1% 8.1% 1%	313 609 82 10
**Education**		
illiterate primary intermediate higher	0.3% 0.5% 78.7% 16%	3 51 798 162
**Records of underlying disease**		
yes no	35.9% 64.1%	364 650
**Relatives had COVID-19**		
yes no	82.6% 16.4%	848 166

The results of the linear regression test in Table 2 showed that there was a significant linear relationship between the variables identity and passive significance; outcomes with relieving; positive regulation and self- blaming; personal controllability and behavioral disengagement; humor and acceptance; therapeutic controllability and active confrontation and behavioral disengagement; coherence of the disease and active confrontation, denial, behavioral disengagement, positive regulation, planning, acceptance, religion, and self-blaming; periodic disease course with denial, acceptance, and religion; emotional representation with denial, behavioral disengagement, relieving, acceptance, and self-blaming; cause of disease with positive regulation and self-blaming. P values are specified in Table 2.

**Table 2:** correlation between perception of disease and coping strategies in COVID-19 patients

Variables	Self-distraction	Active confrontation	denial	Drug use	Supply support	Social support	Behavioral disengagement	Relieving	Positive regulation	Planning	Humor	Acceptance	Religion	Self-blaming
Identity	0.019	-0.164*	-0.018	0.070	0.087	0.042	-0.016	0.231**	-0.107	0.119	-0.035	-0.001	0.124	0.278**
Course of disease	0.150	-0.012	-0.158*	0.046	-0.005	-0.032	-0.060	0.056	0.074	0.111	0.050	0.247**	0.018	-0.36
Outcomes	0.087	-0.042	0.023	-0.026	0.094	0.023	0.099	0.229**	-0.275**	-0.052	− 0.0138	-0.006	0.148	0.231**
Personal controllability	0.021	0.141	-0.003	0.009	0.147	0.048	-0.209**	0.122	0.022	0.003	0.169*	0.249**	0.055	0.080
Therapeutic controllability	-0.003	0.265**	0.011	-0.141	0.110	0.014	-0.251**	0.147	0.123	0.047	0.109	0.100	0.125	-0.110
Coherence of disease	-0.30	0.204**	-0.193*	-0.036	0.026	0.092	0.192*	0.144	0.201**	0.342**	0.050	0.215**	0.203**	-0.279**
Course of disease	-0.015	-0.013	-0.219**	0.111	-0.041	0.001	0.109	0.111	-0.065	0.116	-0.060	0.154*	0.198*	-0.037
Emotional representation	0.073	-0.143	0.191*	-0.042	0.007	-0.052	0.227**	0.367**	-0.151	-0.073	-0.133	-0.283**	0.076	0.398**
Cause of disease	-0.042	-0.097	-0.139	0.049	0.037		-0.003	0.044	0.209**	0.189*	0.147	0.053	-0.073	-0.075
^**^ Significance level > 0.01 ^*^ Significance level > 0.05														

Table 3 shows an inverse significant linear relationship between the variables identity and outcomes; emotional representations and cause of disease and aspects of social support, and a direct significant linear relationship between the variables personal controllability, therapeutic controllability, coherence of disease, and aspects of social support. And the variable periodic disease course, only had a significant relationship with family and friends’ support. The important note of this study was that the time course sub-scale had no significant relationship with any of the social support aspects. Pearson Correlation Coefficient indicates relationship intensity and its plus sign shows a direct relationship and the negative sign shows an inverse one between two variables.

**Table 3:** The correlation between perception of disease and social support in COVID-19 patients

Variables	Support of family	Support of friends	Support of important people
Identity	0.435**	0.484**	-0.395**
Course of disease	0.066	0.014	0.038
Outcomes	0.404**	0437**	-0.376**
Personal controllability	0.272**	0.161*	0.234**
Controllability	0.270**	0.287**	0.237**
Cohesion of disease	0.290**	0.297**	0.313**
Course of disease	0.210**	0.200*	-0.176*
Emotional representation	0.244**	0.199*	-0.228**
Cause of disease	0.360**	0.328**	-0.468**
^**^ Significance level > 0.01 ^*^ Significance level > 0.05			

Table 4 shows that the result of step-by-step multiple regression is related to the analysis of the impact of disease perception and coping strategies on perceived social support in Table 4 in its 7th step. According to the results of the regression, we see that the effect of the variables outcomes, self-blaming, cause of disease, personal controllability, active confrontation, use of relieving and narcotic substances are significant to perceived social support (p < 0.05). Also, the amount of variance explained by the perceived social support calculated by these seven variables was 0.53. According to the sign of the regression coefficient of each of these variables, we find that the effect of the variables of outcomes, self-blaming, cause of disease, use of relieving and narcotic substances on perceived social support is an inverse one, while the effect of personal controllability and active confrontation on perceived social support is a direct one. On the other hand, according to the values of the standard regression coefficients, the most significant effect on perceived social support, in the order of their importance, is done by: outcomes, cause of disease, self-blaming, personal controllability, active confrontation, use of relieving and narcotic substances.

**Table 4:** Disease perception step-by-step regression, coping strategies and perceived social support in COVID-19 patients

Variables	B	Standard error	β	t	Significance level
**Fixed value**	777.4	99.2	-	7.8	0.000
**Outcomes**	-9.7	1.8	-0.3	-5.3	0.000
**Self-blaming**	-18.9	5.6	-0.2	-3.3	0.001
**Cause of disease**	-3.4	1.1	-0.2	-3.7	0.000
**Personal controllability**	7.7	2.2	0.2	3.3	0.001
**Active confrontation**	16.3	7.02	0.1	2.3	0.02
**Drug use**	-26.2	11.4	-0.1	-2.2	0.02
**Relieving**	-13.2	6.5	-0.1	-2.01	0.04
B is the regression slope; β is the standardized regression coefficient; t is the standard characteristic to test the significance of each variable in the prediction of the dependent variable					

Table 5 indicates that step-by-step multiple regression results related to investigating the effect of demographic variables, perception of the disease, coping strategies on perceived social support in COVID-19 patients. According to the results from the regression, the effect of variables of outcomes, self-blaming, education, sex, disease phase, use of relieving and narcotic substances, humor, personal controllability, and the cause of the disease are significant on perceived social support (p < 0.05). Also, the amount of variance explained by these seven variables was 65%. Considering the value of the regression coefficient of each of these variables, we find that the effects of outcomes, self-blaming, sex, use of relieving and narcotic substances, and the cause of the disease on perceived social support is an inverse one, while the effect of variables of education, disease phase, humor, and personal controllability on perceived social support is a direct one. On the other hand, according to the standard regression model, the most important variables that have significant effects on social support, in order of their importance, are: disease phase, drug use, education, self-blaming, humor, relieving, sex, personal controllability, and cause of disease.

**Table 5:** Summary of the step-by-step regression between the effects of the demographic variables, perception of disease, and coping strategies and perceived social support in COVID-19 patients

Variables	B	Standard error	β	T	Significance level	R	R^2^
**Fixed value**	788.2	99.2	-	7.6	0.000		
**Outcomes**	-9.7	1.6	-0.3	-5.3	0.000		
**Self-blaming**	-17.9	4.9	-0.2	-3.6	0.000		
**Education**	26.3	5.8	0.2	4.4	0.004		
**Sex**	-119.6	31.02	-0.2	-3.8	0.000	**0.808**	**0.653**
**Disease phase**	57.5	19.7	0.1	2.9	0.004		
**Drug use**	-32.8	10.2	-0.1	-3.1	0.002		
**Relieving**	-13.5	5.8	-0.1	-2.3	0.02		
**Humor**	14.8	6.1	0.1	2.4	0.01		
**Self-control**	5.2	2.04	0.1	2.5	0.01		
**Cause of disease**	-2.2	1.06	-0.1	-2.1	0.03		
R is the multiple correlation between the examined variables in the model; R^2^ is the squared correlation; B is the regression slope; β is the standardized regression coefficient; t is the standard characteristic to test the significance of each variable in the prediction of the dependent variable							

## Discussion

The aim of this study was to determine the role and relationship between perception of disease, coping strategies, and perceived social support among COVID-19 patients in Khuzestan, Iran in 2020–2021. Recently, researchers have emphasized on paying special attention to providing psychosocial care during COVID-19 prevalence [25–27]. The findings of the present study addressed the necessity of the provision of such services (including paying attention to reactions, active listening, participation in online groups, and practical activities, etc.). Our finding showed that there is a significant correlation between perception of disease, coping strategies, and perceived social support. Lan et al. found that disease perception has an effective role in using coping strategies in breast cancer patients [7]. Also results’ Mariani et al. in Italy were showed that during the quarantine due to COVID-19 pandemic, emotion-focused coping strategies increased anxiety symptoms and depression, probably due to the non-controllable nature of the stressor and the emotional response. They noted that family support played an exclusive role in reducing loneliness [25]. Among COVID-19 patients, perceived social support was inversely related to coping strategies such as self-blame, self-control, and active coping with drug use. In addition, perceived social support and coping strategies in patients were positively correlated with variables such as outcome, cause of illness, personal control, acceptance, and religion. Kandeger et al. were reminded that identifying factors such as perceived social support and coping strategies that can increase individuals’ psychological adjustment and resilience is important to maintaining the physical and mental health of society during the COVID-19 [28]. There are many studies that have researched on the effects of social support and coping strategies on diseases )25, 14), but the COVID-19 disease is an acute infectious disease in which the patient’s condition is completely different and its complications are not yet fully understood. It seems during COVID-19 epidemic quarantines, the focus on feelings of anxiety and depression symptoms appears to increase due to the uncontrollable nature of the stressful event and the high emotional response, decreased family support, and loneliness [26].

In this study, the results of regression showed that there is a significant relationship between outcome variables, self-blame, individual control, active coping, use of drugs, with perceived social support. Comparing the results of various studies in this field shows that in COVID-19 patients, dimension of outcome in perception of disease is important unlike other diseases, especially chronic diseases, in which the dimension of identity is valuable in perceived the disease [8, 12–14]. Because, the disease is life-threatening and causes premature death [27]. Most patients in the present study do not fully understand the nature of the disease and are constantly concerned about their condition. These patients also underestimate their ability to cope with the stress of illness. The feeling of empowerment in people who have more control over their disease, leads them to better control. In general, these people control the disease by relying on a sense of effectiveness in controlling stressful situations, and less than they are, evaluate its negative emotional effects [16]. In general, a positive perception of the disease that is accompanied by an understanding of control and its symptoms will lead to appropriate therapeutic action [10, 26]. As a result, a person with better social support is expected to have a positive perception of the disease using adaptive coping strategies. Maclnnes believes that the interpretation of the symptoms of the disease is due to the formation of perception in the context of nature, controllability and emotions associated with the disease. In the next stage, coping strategies are developed based on these approaches, and in contrast, these coping strategies also affect the perception of the disease [25].

The aim of this study was to determine the relationship between demographic variables, perception of the disease and strategies to cope with perceived social support. Given the value of the regression coefficient of each of these variables, we found that the relationship between variables such as outcomes, self-blame, sex, drug use and the cause of the disease is inverse to perceived social support, while the relationship between education variables, the stage of the disease, humor and self-control over the quality of life is a direct relationship. On the other hand, based on standard regression coefficients, the variables that have a significant relationship with perceived social support in order of importance are: disease stage, substance use, education, self-blame and humor, soothing methods, sex, consequences, personal controllability, and cause of illness. In this study, most of the patients had a moderate level of literacy. Therefore, they did not have enough knowledge to use coping models such as problem solving. However, due to the prevailing local culture, they enjoyed the support of family and friends.

Our findings support the perceived social support interventions in patients for a positive understanding of the disease and applying adaptive coping strategies. Social support from family, friends (p = 0.092) through patient financial support or disease-related information can reduce the burden of patient care costs, reduce stress, and improve disease control. Perceived social support can improve mental adjustment, which will lead to maintaining the patient’s physical and mental health. Improving the patient’s physical and mental health will reduce treatment costs and government, as well as improve the patient’s prognosis [28–30]. The majority of people in Khuzestan are Arab and Lor who often live in extended families. Therefore, among them, family support and maintaining friendly relations between them is of particular importance. The impact of this ruling culture among people is shown in the results of the present study, which shows family and friendly support.

The COVID-19 pandemic is a global crisis that still involves the members of the treatment team, especially nurses who are required to provide 24-hour care. Facing this crisis has increased the workload for them more and more. Therefore, awareness of mental-psychological conditions along with physical problems of the patient can provide better care and satisfaction of the patient, family and even nurses.

## Limitations

The present study was conducted only on COVID-19 patients in Khuzestan province. Therefore, its results cannot be generalized to all patients treated for COVID-19 in other regions. Also, this study was conducted in Iranian population, and using its results in other populations should be done with caution. Considering that the understanding of the disease and the use of coping strategies and social support depends on the culture of the society, therefore, the mentioned strategies may be different from the societies and cultures of other nations. Based on this, it is suggested to evaluate the relationship of these variables in different societies. Another limitation of our study was the requirement to complete the questionnaire. The questionnaire was completed as a self-report. Therefore, the education level and emotional conditions of the participants can affect the accuracy of answering the questions.

## Conclusion

The results of the study showed that the negative perception of the disease is associated with inconsistent coping strategies and perceived social support, and a more adaptive strategy comes with a better perceived social support. A negative perception of the disease can affect seeking support, starting treatment early, and attending hospital care. Recognition of coping strategies and the degree of perception of the disease can be a positive step in the planning of intervention programs to be more positive regarding the disease and its treatment. Given the undeniable role of perceived social support in prediction of patients’ disease perception, this study can be used to plan training programs and effectiveness of interventions and reduce the health care costs imposed on the patient, and society, and a better control over COVID-19. Coping strategies and perceived social support that can improve mental adjustment and resilience are important for maintaining the psychological and physical health in COVID-19 patients. Totally, nurses should be a part of the movement that evaluate the benefits of social support, coping strategy, and perception of the disease in COVID-19 patients and considers these factors in their patient’s care.

## Data Availability

The datasets used during the current investigation are available from the corresponding author on reasonable request.
